# Study on Cavity Filling Defects and Tensile Properties of L-Shaped Profiled Rings

**DOI:** 10.3390/ma17194930

**Published:** 2024-10-09

**Authors:** Tiewen Hao, Junzhe Chen, Tao Zhang, Zhenyang Qin, Yunxin Wu

**Affiliations:** 1 Light Alloy Research Institute, Central South University, Changsha 410083, China; 18698623017@126.com (T.H.); junzhechen2001@outlook.com (J.C.); qinzhenyang2022@163.com (Z.Q.); yxwucsu@163.com (Y.W.); 2 State Key Laboratory of Precision Manufacturing for Extreme Service Performance, Central South University, Changsha 410083, China; 3 Tianjin Heavy Industry Co., Ltd., China First Heavy Industries, Dalian 116600, China

**Keywords:** L-shaped ring, cavity filling defect, ring quality, hardness, tensile properties

## Abstract

Severe cavity filling defects and poor mechanical properties increase the difficulty in the integrated forming of L-shaped profiled rings due to its asymmetrical section geometry. A novel rolling method of a C-shaped ring was proposed in this study, and two symmetric L-shaped rings were prepared simultaneously. A numerical model of C-shaped ring rolling was established, and the cavity filling defects in different directions and the overall forming defect were defined for a qualitative analysis of the geometry’s accuracy. The effect of the rolling parameters on the forming defects and ring quality was investigated. The forming defects increased with an increase in the groove depth ratio as well as decreases in the groove angle and rolling ratio. The feeding strategy with a constant ring growth velocity led to the best geometric accuracy and strain uniformity of the C-shaped rings. Optimized rolling parameters can be acquired by the Box–Behnken optimization method with multi-objective optimization of the rolling stability and ring quality. An experiment of C-shaped ring rolling was successfully prepared, based on the optimized parameters. The hardness distribution on the cross-section was symmetric and uniform. The C-shaped ring showed obvious anisotropy of the tensile properties of the cast ring’s blank, and heat treatment had little effect on the improvement of the isotropy.

## 1. Introduction

The 2219 aluminum alloy (AA2219) has been widely used in the aviation, aerospace, navigation and military industries for its light weight, high plasticity, good weldability and resistance to high and low temperatures [1,2]. L-shaped rings are widely used in various fields, such as the transition ring of rockets, and the flanges and bearings in the energy and power fields [3]. The requirements of high geometric accuracy, isotropic mechanical strength and high fatigue life are essential for the application of L-shaped rings. Ring rolling is the key process for the preparation of the L-shaped rings [4]. However, due to geometric asymmetry in the cross-section of the ring, a bending moment of the ring results from the asymmetrical vertical force on the upper and lower surface of the L-shaped ring. Therefore, L-shaped rings are prone to defects such as unfilling, folding and grooves in the bottom surface during ring rolling [5].

The traditional preparation of a profiled ring consists of two steps: preparation of a ring with rectangular cross-section through the ring rolling process, and then obtainment of the profiled ring through the machining process. However, the material utilization rate is very low, with the removal of large amounts of material; meanwhile, it destroys the metal flow line of the ring, thus decreasing the mechanical properties of the profiled ring. To increase the material utilization and enhance the properties, integrated near-net forming of the profiled ring with high accuracy and excellent mechanical properties was investigated in this study.

A great deal of research has been conducted on the defects produced in the profiled ring rolling process. Cavity filling defects affect the integrity of the geometry on the cross-section of the profiled ring. The integrity of the geometry of the profiled ring is the basic requirement; meanwhile, cavity filling defects also decrease the mechanical properties of the ring, as there is insufficient strain on the defects’ positions. Oh et al. [6] investigated the formation mechanism of defects in L-shaped rings, as well as the effects of processing parameters such as the billet’s geometry, the main roll’s size, the main roll’s speed, the mandrel’s feed speed and working temperature on the defects. Li et al. [7] studied the effects of roll size on the uniformity of strain and the configuration of the ring during the T-shaped ring rolling process, and unreasonable roll sizes (a larger driving roll radius, a smaller mandrel radius or a larger fillet radius at the groove’s entrance) were found to be more prone to inner flaws and surface cracks. Zayadi et al. [8] investigated the effects of some parameters (feed speed, the driving roll’s rotational velocity, the availability of axial rolls) on the cavity filling of single and T-shaped rings, and discovered that axial rolls have a significant effect on the reduction in the cavity filling rate. Hua et al. [9] predicted unreasonable metal streamlines and the uncoordinated growth of three ribs in constraining ring rolling by building analytical models. Tian et al. [10] built a mechanical model to characterize the quantitative relationships among geometric and processing parameters, stress state and the metal flow by using the slab method and the upper bound method.

In order to solve the defects existing in the profiled ring rolling process and to explore the effects of relevant factors on the forming characteristics in the rolling of ring parts, finite element models (FEM) have usually been used. Foraboschi et al. [11] delivered a plain analytical formulation for predicting the ultimate combinations of axial force and bending moment. Hu et al. [12] established the response model via a combination of the response surface method and the FEM, and the cavity filling rate of GH4738 superalloy profile ring could be increased by multi-objective optimization of the geometry and rolling parameters. Banerjee et al. [13] described the stress–strain distribution by simulating the process of ring rolling, and discussed the effect of friction and contact stress on the stress–strain distribution. Zhu et al. [14] explored effects of the dimension factors of the blanks on the profile ring rolling of hard-to-deform materials. Through control of the dimensions and deformation of the ring blank, the strain and deformation distribution can be improved. Qian et al. [15] presented three kinds of rolling methods for the three kinds of groove-section rings and explored the effect of different rolling methods on the geometric accuracy of rolled rings and the material’s flow behavior. Ring rolling with multiple contacts and multiple feeds was suitable for asymmetrical deep-groove rings and cavity filling. Zayadi et al. [16] researched the effects of some parameters (the mandrel’s feed speed, the main roll’s rotational velocity and the guide rolls’ forces) on the geometrical defects. Increasing the feed speed and decreasing the main roll’s rotational velocity both decreased fishtail defects and improved the rings’ geometrical quality. Liang et al. [17] introduced a pulling coefficient to quantitatively describe the pulling effect of the active deformation zone on the passive deformation zone and explored its relationship with the relative groove height of the target rolled ring.

The microstructural evolution plays a vital role in the mechanical properties in the ring rolling process. Zhang et al. [18] researched the microstructure and anisotropy in the tensile strength and tensile creep resistance of an AZ80-Ag alloy rolling ring. Wang et al. [19] studied the microstructural evolution and tempering transformation kinetics of M50 steel during cold ring rolling. Guo et al. [20] investigated the effects of the rolling temperature on the microstructural uniformity and mechanical properties of AA2219 rings. Jiang et al. [21] researched the macrostructure, microstructure and mechanical properties’ evolution during 8Cr4Mo4V steel ring rolling. Hu et al. [22] developed a FEM model to analyze the influence of different rolling passes on the thermal parameters and microstructural evolution during the ring rolling process of a GH4738 superalloy. Han et al. [23] investigated the plastic deformation behaviors and mechanical properties of rolled rings of a 20CrMnTi alloy under different rolling ratios by using the finite element method. Wang et al. [24] researched the interrelationships between the α- and β-phase morphologies, as well as their strength and plasticity properties in tensile tests at room and elevated temperatures in a Ti-6Al-4V alloy processed by ring rolling. Dong et al. [25] conducted a multi-scale simulation about the flow behavior and microstructural evolution of a 2219 aluminum alloy during ring rolling.

The problems of instability and forming defects induced by the asymmetrical rolling force on the upper and lower surfaces of the L-shaped ring have been investigated by many researchers. Previous studies focused on the effect of single parameters on the forming defects, such as the geometric size of the cross-section of the ring blank or L-shaped ring, and the rolling parameters. In fact, the forming defects are related to both the geometry and the rolling parameters. Additionally, the distributions of strain and temperature also affect the rings’ quality as well as the forming defects. Despite the optimization of the ring rolling process of L-shaped rings, rolling instability is quite difficult to solve due to the asymmetrical geometry and rolling force.

In this study, the rolling process of a C-shaped ring was proposed. Two L-shaped rings could prepared simultaneously in the ring rolling process and the rolling stability was expected to be improved due to the symmetric geometry and rolling force of the C-shaped ring. Two L-shaped rings could be obtained by machining the C-shaped rolled ring along the center of the ring’s cross-section. The numerical model of the C-shaped ring was established. The cavity filling defects in different directions and the overall forming defect of the C-shaped ring were defined for a qualitative analysis of the geometric accuracy. The effect of the rolling parameters and the mandrel’s feed strategy on the forming defect and ring quality was investigated. The Box–Behnken method was adopted for optimization of the rolling parameters, and the rolling stability and ring quality were set as the optimization’s objectives. Finally, experiments on C-shaped rings were conducted. The cavity filling defects, hardness distribution on the cross-section and the tensile properties were analyzed.

## 2. Description of the Models

**Figure 1:**
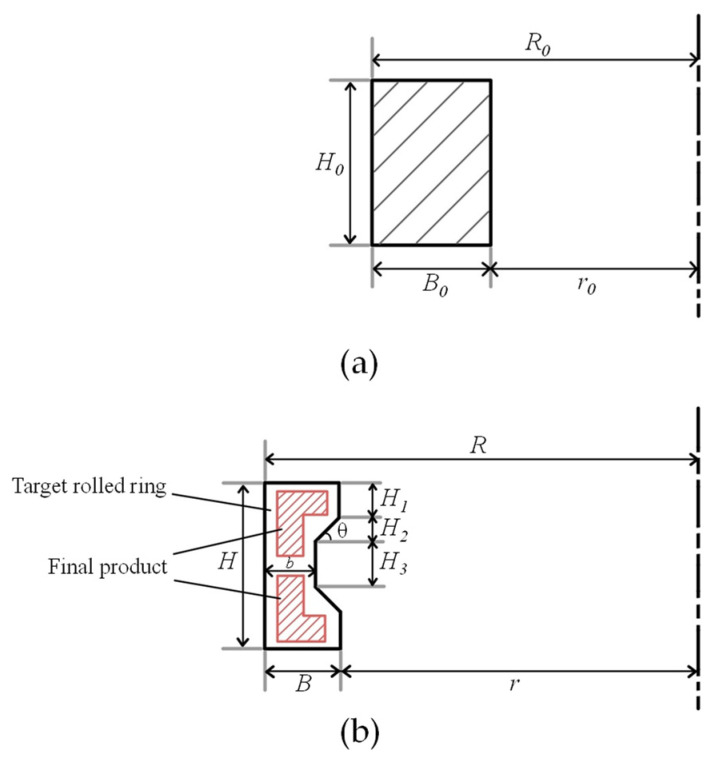
Schematic diagram of (**a**) the ring blank and (**b**) the target C-shaped ring.

As discussed above, the large difference in the dimensions between the upper and lower surfaces of an L-shaped ring result in bending behavior during the ring rolling process, causing severe cavity filling defects and increased difficulty in the integrated forming of the L-shaped ring. Therefore, a C-shaped ring was set as the target in the ring rolling process, and the bending behavior was expected to be improved due to its symmetric geometry and rolling force. As shown in Figure 1, two L-shaped rings can be obtained after the integrated rolling process of the C-shaped ring through the machining process along the center line. The same dimensions between the upper and lower surfaces of the C-shaped ring decreased the bending behavior and improved the stability of the rolling process. The dimensions of the C-shaped ring are shown in Table 1, and a ring blank with a rectangular section was adopted.

**Figure 2:**
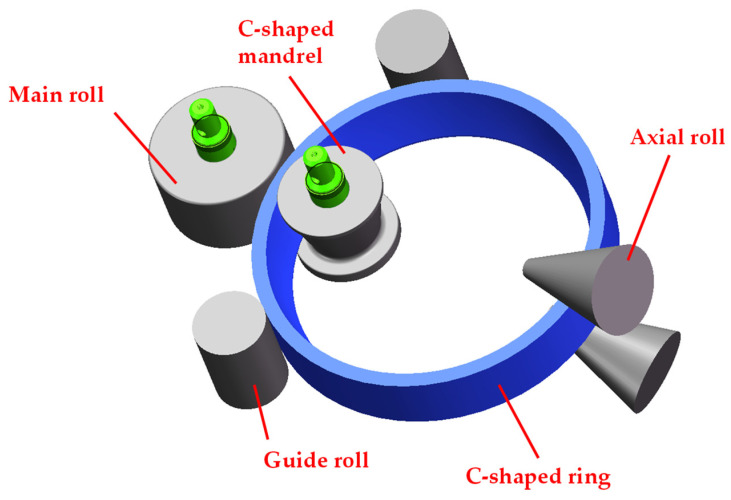
A 3D CAD model of the C-shaped ring rolling process.

The thermo-mechanical coupled finite element model of the C-shaped ring rolling process was established on the basis of ABAQUS v6.14/Explicit software. The main roll, mandrel, guide roll and conical roll were set as rigid bodies. As shown in Figure 2, the material of these rolls was 5CrNiMo with a density of 7.9 g/cm^3^, and the ring was a 2219 aluminum alloy with a density of 2.83 g/cm^3^. The constitutive equation for the 2219 aluminum alloy at elevated temperatures can be referenced in the authors’ previously published article [26].

The ambient temperature was 20 °C, and the initial rolling temperature of the ring was 500 °C. Each roll was sprayed with an emulsion with a temperature of 80 °C. During the ring rolling process, there was heat exchange between the ring and the rolls, and its heat transfer coefficient was 10,000 W·m^2^/K. Meanwhile, there was heat convection and heat radiation between the ring and the environment. The heat convection coefficient was set as 20 W·m^2^/K, and the heat radiation coefficient was 0.7. The friction coefficient between the ring and the guide roll was set as 0.1. As there was strong deformation between the ring and the main roll, mandrel and conical roll, the friction coefficient was set at 0.3. The ring was made of C3D8RT linear hexahedral elements with a grid number of 19,440 and a minimum mesh size of 26 mm. Meanwhile, the ALE adaptive meshing technology was adopted to ensure that the metal material maintained a better mesh state under the severe deformation of the rolling process. The specific rolling parameters are shown in Table 2.

As can be seen from Figure 1, the use of the C-shaped ring enhances the material utilization; meanwhile, it also affects the degree of cavity filling in the ring rolling process. The groove depth and groove angle were key parameters affecting the geometric characteristics of the cross-section. To quantitatively describe the influence of these parameters, the groove depth ratio *K* and the groove angle *θ* were defined, as shown in Equations (1) and (2).

**Figure 3:**
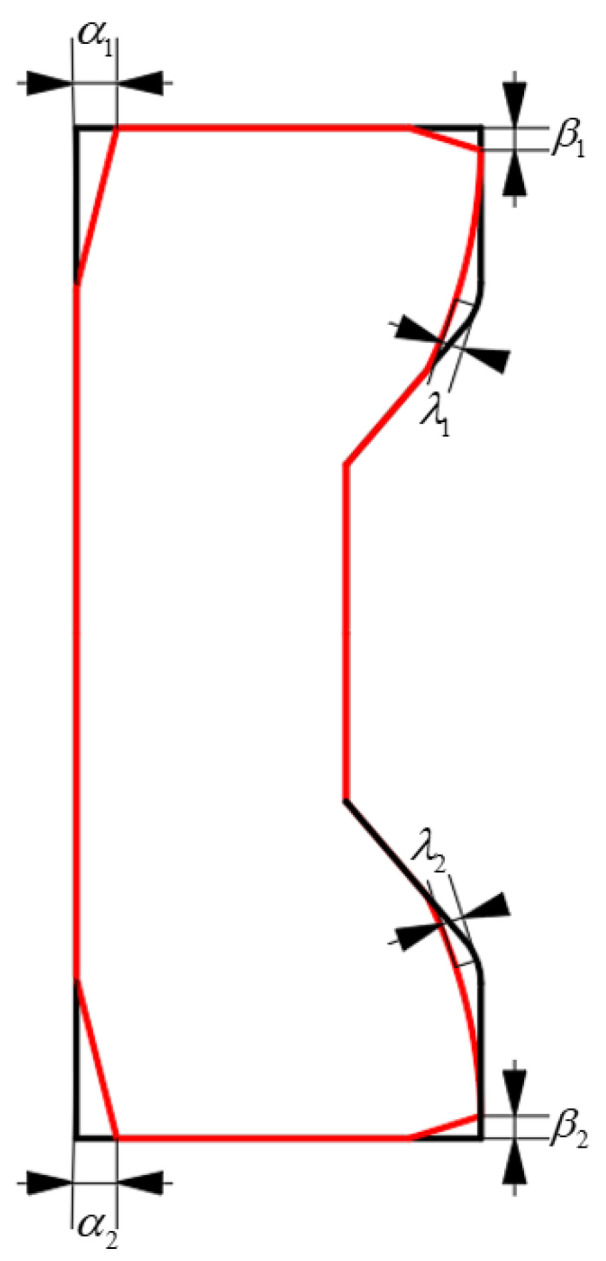
Definition of cavity filling defects in different directions of the ring.

As shown in Figure 3, cavity filling defects may occur in the radial and axial directions on the surfaces as well as the groove corner of the C-shaped ring. The variation in the cavity filling defects in the radial direction, axial direction and groove corner is defined in Equations (3)–(5). To quantitatively describe the effect of the forming parameters on the cavity filling defects, the overall forming defect *δ* was defined as shown in Equation (6) with an equal weight coefficient for the cavity filling defects in different directions

(1) $$K=\frac{B-b}{B},$$

(2) $$\theta =\arctan\left(\frac{H_{2}}{B-b}\right)$$

where *K* is the groove depth ratio, *B* and *b* are the maximum and minimum thickness of the C-shaped section, and *θ* is the groove angle.

(3) $$\alpha =\max(\alpha_{1},\alpha_{2}),$$

(4) $$\beta =\max(\beta_{1},\beta_{2}),$$

(5) $$\lambda =\max(\lambda_{1},\lambda_{2}),$$

(6) $$\delta =\frac{\alpha +\beta +\lambda }{3},$$

where α_1_ and α_2_ are the values of the radial filling defects at the upper and lower surfaces of the section, β_1_ and β_2_ are the values of axial filling defects at the upper and lower surfaces, and λ_1_ and λ_2_ are the values of defects at the groove corners. They are obtained by solving for the distance between the filling defect and the corresponding verge.

Cavity filling defects affect the geometry accuracy of the profiled ring. Meanwhile, rolling force fluctuation determines the rolling stability, and the distributions of strain and temperature affect the quality of the profiled ring. Therefore, the standard deviation of the rolling force (SDF) was defined to describe the rolling stability at the stage of stable rolling, as shown in Equation (7). The uniformity of strain and the temperature distribution of the profiled ring significantly affect the mechanical properties of the ring. The equivalent standard deviation of strain (SDP) and standard deviation of temperature (SDT) are defined as shown in Equations (8) and (9).

(7) $$\mathrm{SDF}=\sqrt{\frac{\sum_{i=1}^{N}{\left(F_{i}-F_{a}\right)}^{2}}{N}},$$

(8) $$SDP=\sqrt{\frac{\sum_{i=1}^{N}{\left(PEEQ_{i}-PEEQ_{a}\right)}^{2}}{N}},$$

(9) $$SDT=\sqrt{\frac{\sum_{i=1}^{N}{\left(T_{i}-T_{a}\right)}^{2}}{N}},$$

where *PEEQ*_i_, *T*_i_ and *F*_i_ are the equivalent plastic strain, temperature and rolling force of node *i*, respectively; *N* is the number of nodes in the ring; and *PEEQ*_a_, *T*_a_ and *F*_a_ are the average equivalent plastic strain, temperature and rolling force of all nodes in the ring.

## 3. Results and Discussion

In the rolling process of profiled rings, the groove depth ratio, groove angle and rolling ratio change the geometry of the cross-section of the ring blank and the profiled ring, thus affecting the metal flow and cavity filling behavior on the cross-section of the ring. Additionally, the distributions of the strain and temperature during the rolling process also affect the rings’ quality, which vary under different rolling parameters.

### 3.1. Effect of the Groove Depth Ratio

**Figure 4:**
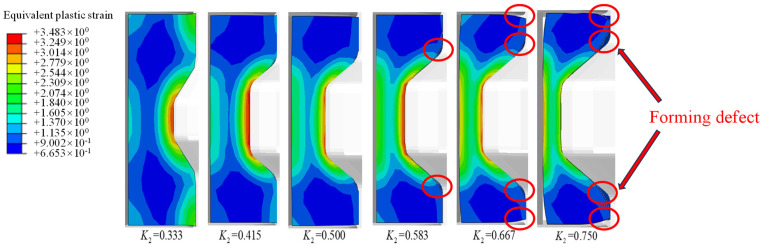
Effect of the groove depth ratio *K* on the cavity filling defects.

Figure 4 shows the influence of different groove depth ratios on the cavity filling defects with a groove angle of 33.7°. There are no obvious forming defects at a small groove depth ratio, while the forming defects in the radial and axial directions as well as the groove corner can be easily seen when the groove depth ratio increases to 0.667. The bending behavior is quite severe with the groove depth ratio of 0.75, which has difficulty satisfying the requirements of dimensional accuracy of the profiled ring.

**Figure 5:**
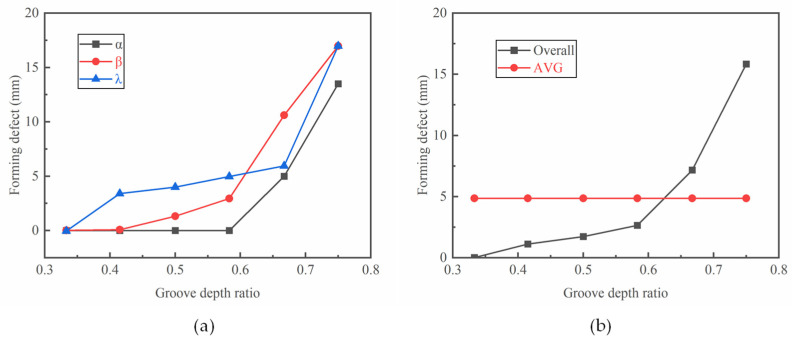
Effect of the groove depth ratio K on (**a**) the cavity filling defects in different directions and (**b**) the overall forming defect.

Figure 5a shows the cavity filling defects in different positions of the ring with variations in the groove depth ratio. There are no radial filling defects with the groove depth ratio of 0.583, while they increase significantly with further increases in the groove depth ratio. There is only a slight increase in the axial filling defect with groove depth ratios smaller than 0.583; it increases sharply with further increases in the groove depth ratio. Different from the variation in radial and axial filling defects, the filling defects start to appear at the groove corner under small groove depth ratios, indicating the difficulty of the metal flow at these corners. It can be seen that the cavity filling defects in different positions all increase with an increased groove depth ratio, and the value of the defect in the groove corner is larger than that in the radial and axial directions. On the basis of Equation (6), the overall forming defect can be calculated, as shown in Figure 5b. The forming defect is positive related to the groove depth ratio. The increment in the forming defects is small with groove depth ratios ranging from 0.333 to 0.583, while it significantly increases with groove depth ratios over 0.583. The average value of the forming defects under different groove depth ratios (Figure 5b: AVG) was also calculated. The forming defect exceeds the average value at a groove depth ratio of 0.667.

**Figure 6:**
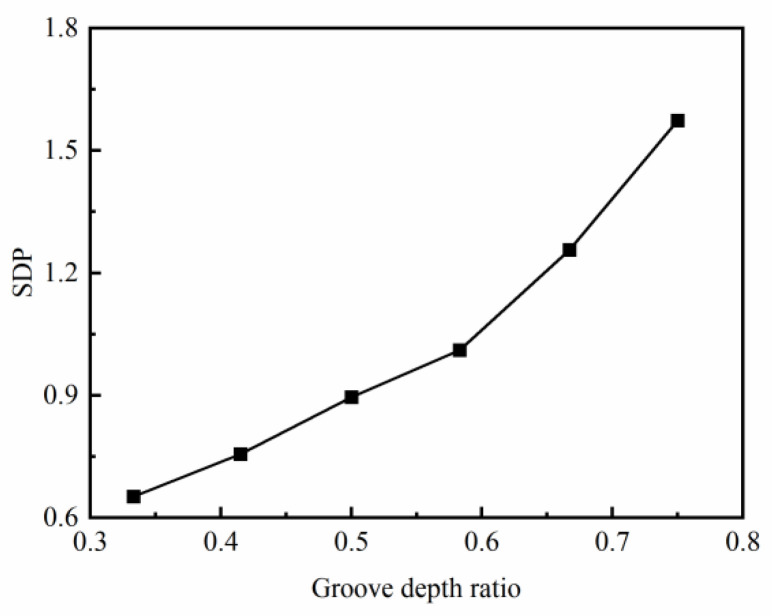
Effect of the groove depth ratio K on SDP.

The SDP on the cross-section of the ring increases almost linearly with an increase in the groove depth ratio, as shown in Figure 6. Larger groove depth ratios increase the difficulty of the cavity filling process. There is a large difference in the deformation resistance of the metal in different directions, which increases the heterogeneity of the strain distribution.

The variation in the groove depth ratio also affects the material utilization of the profiled ring compared with that of the ring with a rectangular cross-section. The material utilization rate increases from 16.3% to 36.7% with groove depth ratios ranging from 0.333 to 0.75. As discussed above, the forming defects and SDP both increase with an increased groove depth ratio. The forming defects are smaller than the average value and the SDP is also at a small value with a groove depth ratio less than 0.667; meanwhile, the material utilization rate is 32.6%. Therefore, the groove depth ratio should be less than 0.667 for C-shaped rings, considering the forming defects and material utilization.

### 3.2. Effect of the Groove Angle

**Figure 7:**
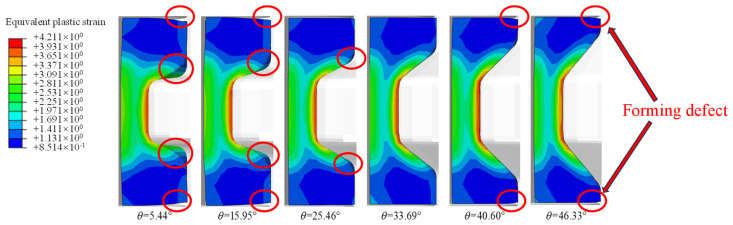
Effect of the groove angle θ on the forming defects.

Figure 7 shows the influence of different groove angles on the cavity filling of the rings with a groove depth ratio of 0.583. There are radial and axial cavity filling defects and an obvious bending phenomenon at the groove corner of the ring at small groove angles. During the ring rolling process, there is radial metal flow, with a simultaneous feed motion of the mandrel and the axial metal flow during the cavity filling process. According to the principles of metal forming, the deformation resistance is largest for the metal flow in the orthogonal directions. With the decrease in the groove angle, the joint angle between the upper part and center part of the C-shaped ring is close to 90°. The difficulty during cavity filling leads to the forming defects. With an increase in the groove angle, the cavity defects at different positions as well as the degree of bending behavior are obviously reduced. When the groove angle increases to 33.69°, the cavity filling is basically complete. However, it is worth noting that an axial filling defect appears again with further increases in the groove angle.

**Figure 8:**
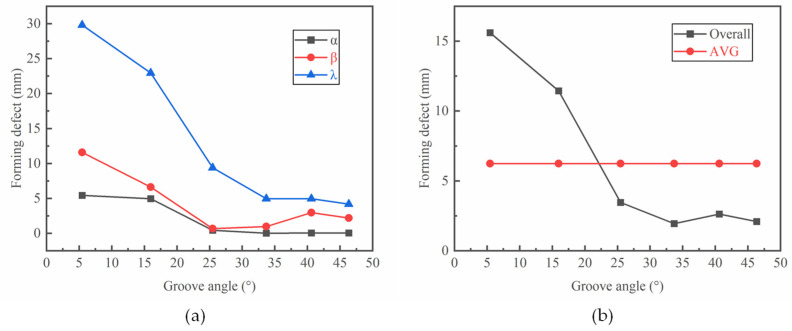
Effect of the groove angle θ on (**a**) cavity defects in different directions and (**b**) the overall forming defect.

As can be seen from Figure 8, with an increased groove angle, the cavity filling defects in radial direction and groove corner first decrease and then remain unchanged. The axial filling defects first decrease and then show a fluctuation with increased groove angles. The values of the radial and axial filling defects almost decrease to zero with a groove angle of 25.46°. The value of filling defects at the groove corner is much larger than that in the radial and axial directions of the ring. The maximum value of filling defects at the groove corner is 30 mm, while it is 5.32 mm and 11.63 mm in the radial and axial directions, respectively. The variation in filling defects at the groove corner is sensitive to the change in the groove angle. It decreases from 30 mm to below 5 mm with groove angles ranging from 5.44° to 33.69°. The average value of the forming defect under different groove angles (Figure 8b: AVG) was also calculated. The overall forming defect is smaller than the average value when the groove angle is larger than 25.46°.

**Figure 9:**
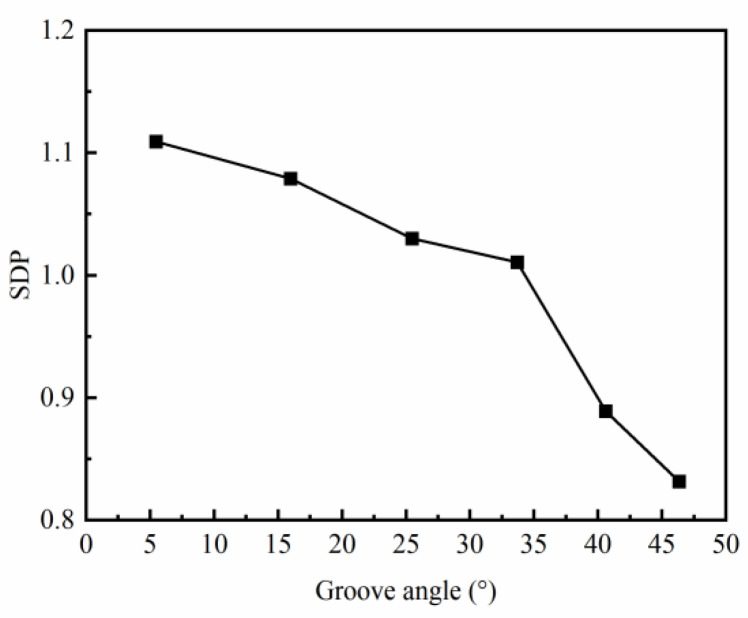
Effect of the groove angle θ on SDP.

The SDP of the C-shaped ring first decreases slightly and then reduces obviously with an increase in the groove angle (Figure 9). As discussed above, a smaller groove angle leads to a larger difference between the radial and axial deformation resistance, thus resulting in obvious anisotropy of the metal’s flow behavior and the inhomogeneity of the strain distribution. Additionally, the groove angle also affects the material utilization rate, which increases from 20.7% to 33.7% when the groove angle increases from 5.44° to 46.33°, compared with the ring with a rectangular cross-section. Therefore, a large groove angle is recommended for the C-shaped ring, considering the forming defects and material utilization.

### 3.3. Effect of the Rolling Ratio

The rolling ratio is defined as the rectangular cross-section area of the ring blank to the C-shaped cross-section area of the profiled ring. During the ring rolling process, the height of the ring blank equals that of the C-shaped ring, and conical rolls are adopted for the flattening of the flash-generated feed motion of the mandrel. On the basis of the constant volume in the plastic forming process, the rolling ratio determines the geometric size of the ring blank. The rolling ratio is the key parameter for the strain of the ring. Large rolling ratios lead to a great thickness of the ring blank, which increases the inhomogeneity of the strain of the ring and the capacity of the rolling machine. Small rolling ratios increase the diameter of the ring blank and increase the difficulty in the preparation of the ring blank. Meanwhile, small rolling ratios will shorten the rolling time, and forming defects may occur due to insufficient time for the cavity filling process.

**Figure 10:**
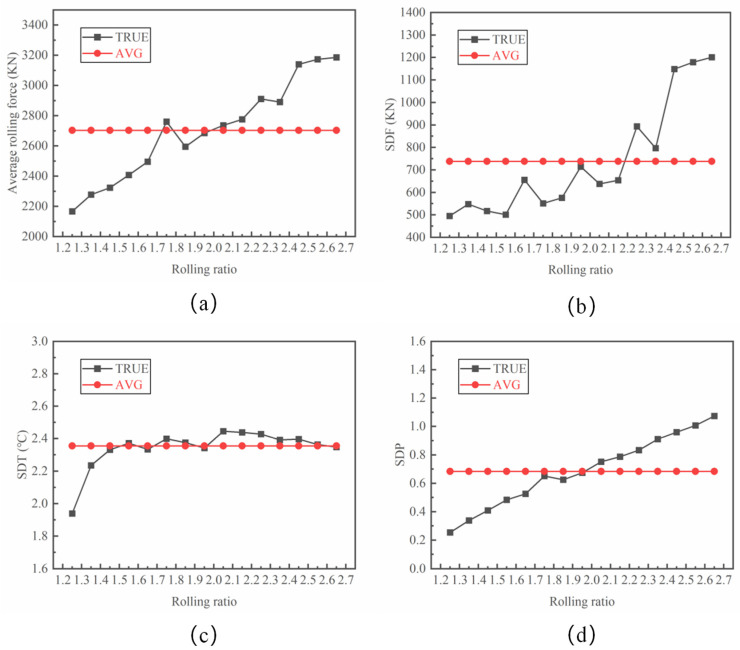
Effect of the rolling ratio on (**a**) the average rolling force, (**b**) SDF, (**c**) SDT and (**d**) SDP.

As shown in Figure 10, the average rolling force increases with an increase in the rolling ratio, despite the fluctuations. A larger initial thickness resulting from the larger rolling ratio increases the rolling force during the rolling process. The SDF also rises with fluctuations with an increase in the rolling ratio, indicating the increased rolling instability under a large rolling ratio. Meanwhile, the average value of the rolling force and SDP for the different rolling ratios (Figure 10: AVG) were also calculated. It can be seen that the value of SDF is below the average level with the rolling ratio at [1.25,1.95]. The SDP increases linearly with an increased rolling ratio, while the SDT first increases and then shows obvious fluctuations. It is interesting that the values of SDP and SDT are below their average values with rolling ratios ranging from 1.25 to 1.95.

To study the effect of the rolling ratio on the geometric accuracy of the C-shaped ring, the error in the outer diameter and the ovality of the rolled ring are defined in Equations (10) and (11), respectively.

(10) $$e_{D}=\frac{D-D^{\prime }}{D}\times 100\%,$$

(11) $$e_{M}=R_{\max}-R_{\min},$$

where *e_D_* is the error in the outer diameter, and *e_M_* represents the ovality of the rolled ring. *D* and *D*′ are the targeted and instantaneous outer diameter after rolling process, respectively, and *R*_max_ and *R*_min_ are the maximum and minimum radius of the ring after the rolling process.

**Figure 11:**
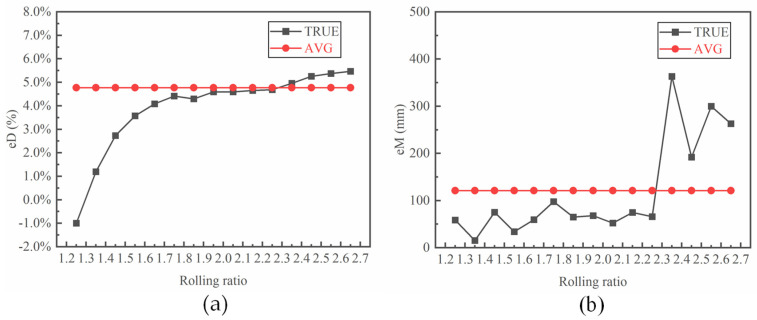
Effect of the rolling ratio on (**a**) the error in the outer diameter and (**b**) the ovality of the ring.

The error in the outer diameter first increases sharply and then increases slightly with an increase in the rolling ratio without considering the final rounding stage of the ring. The outer diameter error is negative with a rolling ratio of 1.25, indicating that the outer diameter of the ring exceeds the targeted size, as well as the failure of the rolling process. The average value of *e_D_* and *e_M_* (Figure 11: AVG) for the different rolling ratios was also calculated. The value of the outer diameter error is below the average value when the rolling ratio changes from 1.35 to 2.25. The ovality shows a slight fluctuation with rolling ratios from 1.25 to 2.25, and then it increases significantly with further increases in the rolling ratio. As discussed above, a large rolling ratio increases the rolling instability, thus resulting in severe ovality of the ring.

According to a comprehensive consideration of the distributions of strain, temperature and rolling force, as well as the geometric accuracy of the ring, the rolling ratio of 1.95 was selected in this study.

### 3.4. Effect of the Mandrel’s Feed Strategy

For the ring rolling process, the mandrel’s motion mainly consists of three feed strategies: a constant radial feed rate (Strategy I), a constant ring growth velocity (Strategy II) and a segmented feeding strategy with constant feed rate first and then a constant growth velocity (Strategy III). The average values of the rolling force, temperature and equivalent plastic strain derived from ABAQUS v 6.14 software for the entire rolling process were calculated. As shown in Table 3, Strategy I shows the smallest degree of fluctuation in the rolling force, indicating that the constant feed rate of the mandrel is beneficial for improvement of the stability in the rolling process. However, the mandrel’s feed rate needs to be adjusted instantaneously to maintain a constant ring growth velocity in Strategy II, thus resulting in large fluctuations in the rolling force. For the segmented feeding strategy, although the average rolling force in the entire rolling process is the smallest, it causes the largest rolling force fluctuations, meaning that the rolling process of C-shaped ring is most likely to be unstable under this strategy.

It can be seen from Table 4 that the average temperature and SDT vary little under different feed strategies. It means that the heat generated via plastic deformation and the heat dissipated through heat exchange almost reach a balanced condition after different feed strategies.

Strategy I leads to the largest average equivalent plastic strain and SDP (Table 5). The constant feed rate of the mandrel contributes to the accumulated strain of the ring; meanwhile, the strain on the surface is larger than that on the center part of the ring during the rolling process. As a result, the constant feed rate accelerates the inhomogeneity of strain. During Strategy II, the feed rate decreases gradually during the constant growth of the ring, thus decreasing the average equivalent plastic strain. Additionally, the feed rate coordinates with the ring growth, which is beneficial for decreasing the strain gap between the surface and the center part of the ring, which increases the strain uniformity of the ring.

**Figure 12:**
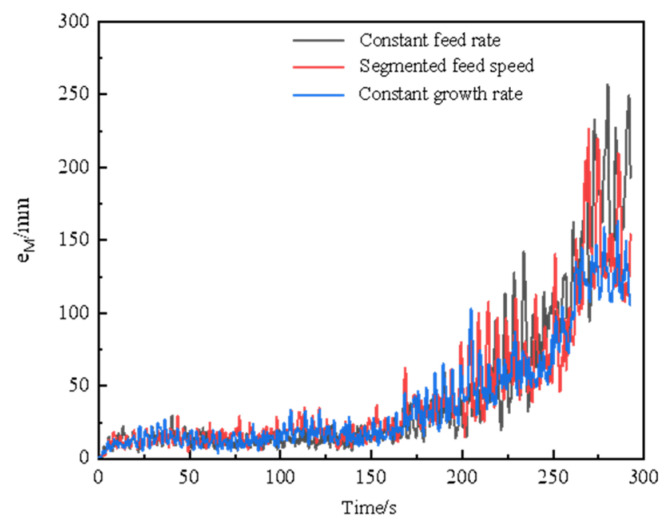
Effect of the mandrel’s feed strategy on the ovality of the ring.

The ovality of the ring first keeps almost stable and then increases sharply during the three strategies, as shown in Figure 12. It can be seen that the value of ovality and its fluctuation in Strategy II are smaller than those in Strategy I and Strategy III. Above all, the feed strategy with a constant ring growth velocity is recommended in this study for high geometric accuracy and strain uniformity, as well as a stable rolling process.

### 3.5. Optimization of the Rolling Parameters

In addition to the geometric parameters discussed above, the rolling parameters (the main roll’s rotation speed, the mandrel’s radius and the ring growth velocity) affect the distributions of strain, temperature and rolling stability. A Box–Behnken response surface was adopted in this study. The SDF, SDP and SDT were set as the optimization targets, and the rolling parameters were the input variables. According to the production experience of the actual rolling process, the range of variation of the parameters is described as follows: the mandrel’s radius is 200–260 mm, the ring growth velocity is 3–5 mm/s and the main roll’s rotation speed is 1–1.7 rad/s. Table 6 shows the results of the Box–Behnken optimization method. The relationships among SDF, SDP, SDT and the rolling parameters were fitted as shown in Equations (12)–(14).

(12) $$\begin{array}{l} SDF\left(A,B,C\right)=7875190-55602.882A-1787350B-1338540C+17589.933AB\\ +122.781A^{2}-19801.78B^{2}+401304C^{2}-39.409A^{2}B \end{array},$$

(13) $$\begin{array}{l} SDP\left(A,B,C\right)=1.903-7.211\times 10^{-3}A-2.466\times 10^{-1}B+8.572\times 10^{-1}C\\ -2.4\times 10^{-5}AB-8.4\times 10^{-5}AC-3.396\times 10^{-2}BC+1.1\times 10^{-5}A^{2}+2.017\times 10^{-2}B^{2}\\ -1.507\times 10^{-1}C^{2} \end{array},$$

(14) $$SDT\left(A,B,C\right)=3.008+1.742\times 10^{-3}A-9.747\times 10^{-2}B-2.976\times 10^{-1}C,$$

where A is the mandrel’s radius, B is the ring growth velocity and C is the main roll’s rotation speed.

Equations (12)–(14) only show the relationship between a single target and the rolling parameters. There may be an interaction among the three targets; meanwhile, the rolling stability and distributions of strain and temperature both affect the rings’ quality. Therefore, it is necessary to carry out multi-objective optimization in order to improve the rolling stability and quality of C-shaped rings, and to optimize the uniformity of the deformation and the overall temperature field. In this study, the ideal point method was used for multi-objective optimization, and SDX* = (*SDF*_min_, *SDP*_min_, *SDT*_min_) was chosen as the ideal point. The optimization model is shown in Equation (15), with the minimum value of the ideal point. Based on the data in Table 6 and the ideal point method, the optimized rolling parameters are described as follows: the mandrel’s radius is 260 mm, the ring growth velocity is 5 mm/s and the main roll’s rotation speed is 1.668 rad/s.

(15) {minSDX(A,B,C)=[(SDF(A,B,C)−SDFmin)2+(SDP(A,B,C)−SDPmin)2+(SDT(A,B,C)−SDTmin)2]12s.t.200≤A≤2603≤B≤51≤C≤1.7,

## 4. Experiments

As discussed above, a C-shaped ring can be rolled by a ring blank with a rectangular cross-section. Experiments on an integrated C-shaped ring rolling process were conducted. The groove depth ratio and groove angle of the C-shaped ring were 0.58 and 35.5°, respectively. The rolling parameters were set as follows, based on the optimized results in Section 4: rolling ratio, 1.95; mandrel’s radius, 260 mm; ring growth velocity, 5 mm/s; main roll’s rotation speed, 1.668 rad/s. C-shaped mandrel molds made of 5CrNiMo were designed, based on the geometry of the C-shaped ring. The material of the ring blank was 2219 aluminum alloy after the casting process, and the initial rolling temperature was 480 °C. The mandrel’s feed strategy with a constant growth velocity of the ring was adopted. Emulsion lubrication was applied during the ring rolling process to avoid abnormal temperature increases.

A portion of the C-shaped ring was cut through the tangential direction, and the forming defects and the hardness distribution on the cross-section were analyzed. Large amounts of cylindrical samples with a diameter of 10 mm were extracted from the cross-section, and the hardness test was conducted with a load of 200 g and a holding time of 15 s. To study the tensile properties and the anisotropy of the C-shaped ring, tensile coupons in the radial, axial and tangential directions were extracted, and a tensile test was conducted with a speed of 1 mm/min. The 2219 aluminum alloy is a kind of heat-strengthened alloy, and a solution and aging treatment was applied to the material. The procedures for heat treatment were as follows: it was first treated at 535 °C for 3 h and quenched in water immediately, then it was aged at 155 °C for 6 h, followed by furnace cooling. The hardness distribution and the tensile properties after heat treatment were also investigated.

**Figure 13:**
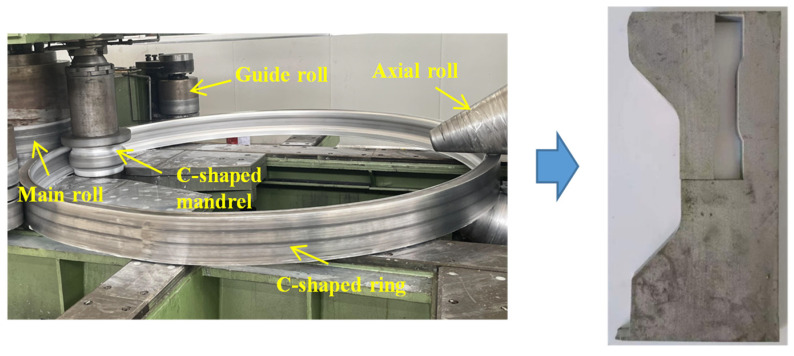
Cavity filling on the cross-section of the C-shaped ring.

Figure 13 shows the cavity filling on the cross-section of the C-shaped ring. On the basis of the optimized parameters, a C-shaped ring was successfully prepared from the ring blank with a rectangular cross-section. It can be seen that there is no bending behavior of the ring due to the symmetrically vertical force from the upper and lower axial rolls. There is a flash with a length of about 7 mm on the lower surface of the C-shaped ring, as there is strong contact friction between the lower surface of the ring and the worktable.

**Figure 14:**
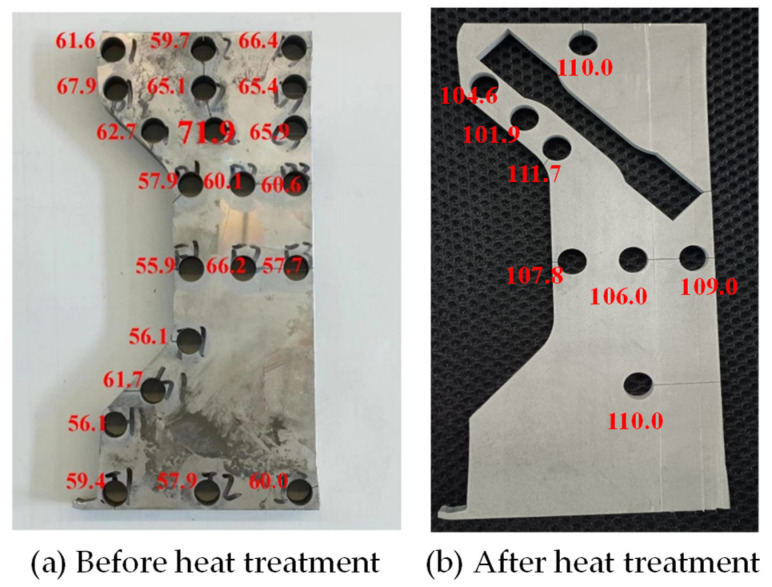
Hardness distribution on the cross-section of a C-shaped ring.

Figure 14 shows the Vickers hardness distribution of the C-shaped ring, measured by the KHVS-1000MT microhardness tester (ATM Qness GmbH, Mammelzen, Germany). The difference in hardness on the cross-section of the ring is small, indicating a near-uniform distribution of the hardness. The average hardness on the upper part of the ring is slightly larger than that on the lower part. The flash resulting from the radial feed motion of the mandrel is mainly generated on the upper surface of the ring due to the direct contact between the lower surface of the ring and the worktable. The flash generated in the radial deformation zone is flattened by the upper axial roll, thus causing larger deformation and greater hardness on the upper part of the ring. The hardness at the straight line on the center part of the C-shaped surface is smaller than that at other positions. The deformation resistance is large at the straight line on the C-shaped surface, thus increasing the difficulty in the metal flow at these positions; as a result, the strain at the straight line is smaller than that at other positions. The heat treatment significantly increases the hardness, and there is still a small difference in the hardness at different positions, which is similar to the distribution before the heat treatment.

Table 7 shows the tensile properties of the C-shaped ring. There is small difference in the the UTS and YS in the tangential and axial directions of the ring, while the radial coupons show the smallest strengths before heat treatment. The elongation in the tangential direction is the largest, with a value of 15.97%, compared with the other two directions. The metal flow in the tangential direction is the main deformation characteristic of the ring rolling process, and the grains are elongated in the tangential direction, thus significantly increasing the ductility. The elongation in the axial direction almost equals that in the radial direction.

The heat treatment obviously increases the strength of the ring due to the precipitation of the strengthening phases, as shown in Table 8. The elongation in the tangential and axial directions of the ring also increases after heat treatment, while the radial ductility sharply decreases. This may be explained as follows. There are severe micro-segregation and coarse second-phase particles during the cast process; meanwhile, the ring blank is made of the cast 2219 alloy. The heat treatment is beneficial for elimination of the micro-segregation and fragmentation of the coarse second-phase particles, thus contributing to enhancement of the strength and improvements in ductility. As for the obvious decrease in the radial elongation after heat treatment, there may be an aggregation of second-phase particles in the radial tensile coupons, which has a detrimental effect on the ductility.

The anisotropy in three orthogonal directions (ATOD) was defined for a qualitative analysis of the anisotropy of the C-shaped ring, as shown in Equation (16). The value of ATOD on the strength is small before the heat treatment, while the anisotropy of elongation still exists due to the deformation characteristics of the ring rolling process, as shown in Table 9. The heat treatment slightly increases the values of ATOD on UTS, illustrating that the heat treatment is not effective in increasing the isotropy of the tensile properties of the ring. Therefore, a multi-forging process is recommended for the preparation of the ring blank, as it is useful for fragmentation of the coarse second-phase particles.

(16) $$\mathrm{ATOD}=\frac{2X_{max}-X_{mid}-X_{min}}{2X_{max}}\times 100\%$$

where *X*_max_, *X*_mid_ and *X*_min_ are, respectively, the maximum, median and minimum values of the parameters in the radial, axial and tangential directions of the ring.

## 5. Conclusions

1. Integrated rolling of a C-shaped ring was proposed in this study, and two vertical symmetric L-shaped rings were prepared simultaneously. The symmetrical geometry of the C-shaped ring was effective in decreasing the severe forming defect and bending behavior of the L-shaped ring.
2. The cavity filling defects in different directions of the C-shaped ring were defined, and the overall forming defect in relation to the forming parameters was also analyzed. The forming defects increased with an increase in the groove depth ratio as well as decreases in the groove angle and rolling ratio. When the groove depth ratio was greater than 0.667 or the groove angle was less than 15.95°, the filling defect coefficient was greater than the average value. The rolling ratio of 1.95 was adopted, considering the cavity filling defects and preparation of the ring blank.
3. The effect of the forming parameters on the rolling stability as well as the distributions of temperature and strain were investigated, and optimized parameters were obtained by the Box–Behnken optimization method.
4. A C-shaped ring was successfully prepared from the ring blank with a rectangular cross-section. The hardness distribution was almost uniform and there was almost no bending behavior in the cross-section of the C-shaped ring. The hardness of the ring increased from 61.73 HV to 107.88 HV after heat treatment.
5. The C-shaped ring showed larger tangential elongation than other two directions. Heat treatment was effective in enhancing the strength, while it had little effect on improving the isotropy of the tensile properties. Therefore, a multi-forging process is recommended for preparation of the ring blanks.

## Figures and Tables

**Table 1:** Dimensions of the C-shaped rolled ring, 3.35 m in diameter.

R (mm)	r (mm)	H (mm)	H_1_ (mm)	H_2_ (mm)	H_3_ (mm)	b (mm)
1675	1495	450	80	70	150	[45,130]

**Table 2:** Parameters for ring rolling.

Parameter	Value
Main roll diameter, mm	850
Guide roll diameter, mm	350
Rotation speed of the main roll, rad·s^−1^	1~1.7
Axial roll taper angle, °	35
Axial roll diameter on the big end, mm	560
Initial rolling temperature, °C	500
Environment temperature, °C	20
Heat transfer coefficient between ring and rolls, W/(m^2^·K)	10,000
Heat transfer coefficient between ring and environment, W/(m^2^·K)	20
Heat emissivity	0.7

**Table 3:** Average rolling force and SDF under three strategies.

Strategy	Average Rolling Force (t)	SDF (t)
Strategy I	259.0	82.0
Strategy III	249.3	89.8
Strategy II	260.8	88.4

**Table 4:** Average temperature and SDT under the three strategies.

Strategy	Average Temperature (°C)	Maximum Temperature Difference (°C)	SDT (°C)
Strategy I	481.97	11.71	2.623
Strategy III	481.59	11.57	2.620
Strategy II	481.46	11.59	2.622

**Table 5:** Average equivalent strain and SDP under three strategies.

Strategy	Average Equivalent Plastic Strain	Maximum Difference in Equivalent Plastic Strain	SDP
Strategy I	1.817	4.123	0.911
Strategy III	1.807	4.067	0.887
Strategy II	1.797	4.061	0.882

**Table 6:** Average equivalent strain and SDP under three strategies.

Number	Mandrel’s Radius (mm)	Ring Growth Velocity (mm/s)	Main Roll’s Rotation Speed (rad/s)	SDF (N)	SDP	SDT (°C)
1	230	4	1.35	883,569.081	0.882	2.622
2	200	4	1.7	847,368.318	1.040	2.451
3	230	5	1.7	840,037.271	0.853	2.420
4	200	4	1	1,036,580.269	0.827	2.666
5	230	4	1.35	883,569.081	0.882	2.622
6	260	4	1.7	786,358.843	0.918	2.571
7	230	5	1	1,016,194.043	0.674	2.636
8	200	5	1.35	864,227.950	0.847	2.463
9	260	3	1.35	832,857.270	0.977	2.791
10	260	4	1	935,131.056	0.710	2.765
11	230	3	1.7	797,789.692	1.117	2.586
12	260	5	1.35	760,009.275	0.724	2.554
13	230	4	1.35	883,569.081	0.882	2.622
14	230	4	1.35	883,569.081	0.882	2.622
15	200	3	1.35	872,497.688	1.102	2.683
16	230	4	1.35	883,569.081	0.882	2.622
17	230	3	1	997,687.382	0.891	2.794

**Table 7:** Tensile properties before heat treatment.

Direction	Number	YS/MPa	UTS/MPa	EI/%
Tangential	1–1	89.7	182.3	16.8
	1–2	88.9	181.0	15.8
	1–3	90.3	178.1	15.3
	Average	89.63 ± 0.57	180.47 ± 1.76	15.97 ± 0.62
Radial	2–1	84.3	159.6	14.2
	2–2	83.6	163.4	14.6
	2–3	85.8	162.1	14.0
	Average	84.57 ± 0.92	161.70 ± 1.58	14.27 ± 0.25
Axial	3–1	94.3	176.6	14.6
	3–2	94.6	178.5	14.3
	3–3	94.8	174.9	14.0
	Average	94.57 ± 0.21	176.67 ± 1.47	14.30 ± 0.2

**Table 8:** Tensile properties after heat treatment.

Direction	Number	YS/MPa	UTS/MPa	EI/%
Tangential	1–1	197.8	369.0	22.4
	1–2	198.6	366.1	20.8
	1–3	200.6	369.5	22.8
	Average	199.00 ± 1.18	368.20 ± 1.50	22.00 ± 0.86
Radial	2–1	196.4	331.2	9.9
	2–2	195.7	321.3	8.2
	2–3	203.2	327.5	8.3
	Average	198.43 ± 3.38	326.67 ± 4.08	8.80 ± 0.78
Axial	3–1	203.5	379.3	16.9
	3–2	211.6	383.3	16.2
	3–3	202.1	374.3	17.1
	Average	205.73 ± 4.19	378.97 ± 3.68	16.73 ± 0.39

**Table 9:** Coefficients of ATOD before and after heat treatment.

	Before Heat Treatment	After Heat Treatment
ATOD_YS_	4.26%	3.00%
ATOD_UTS_	5.01%	9.14%
ATOD_EI_	17.28%	43.94%

## Data Availability

The original contributions presented in the study are included in the article, further inquiries can be directed to the corresponding author.
